# Genito-Urinary Diseases

**Published:** 1904-11-05

**Authors:** 


### Progress in Medicine and Surgery.

### GENITO-URINARY DISEASES.

(*Concluded from page* 71.)

**The Kollmann Five-glass Test in Urethritis.**—G. T. Mandorff[4] has written on this subject. He agrees with the custom of genito-urinary specialists of making urethroscopic examinations in nearly every case of urethral disease, but believes that in a certain proportion of cases such examination cannot be made—for example, in suspected tubercular disease, acute urethritis, stricture, and when there is an abnormally small meatus. Kollmann employs the five-glass test as an additional aid to urethroscopy. The writer recommends its use, not as the equal of urethroscopy, but as an aid to the general practitioner. The principle of the procedure is to thoroughly cleanse the anterior urethra, and then allow the patient to urinate into three glasses. The patient is instructed to call at the office early and to retain the night's urine. A catheter of hard rubber, with the eye close to the end, is then passed to the external sphincter, and with this, using a six-ounce syringe, the anterior urethra is thoroughly washed out and the washings collected in the first urine glass. The irrigation is carried out until the escaping fluid is free from shreds and mucus. For this 10 to 15 irrigations may be required. When it is certain that all shreds have been removed from the anterior urethra he collects the fluid of the last irrigation in the second urine glass, the so-called "control-glass." The patient then micturates successively into three glasses, viz., glasses No. 3, 4, and 5. The contents of glass No. 3 are to be regarded as doubtful, but shreds in glasses No. 4 or 5 signify pathological changes in the posterior urethra, bladder, and possibly kidneys. The test is completed by making microscopical examinations of the shreds. Those from the anterior urethra are composed of long twisted threads or rolls of epithelial cells, pus cells, and mucus. The epithelial cells are usually of the flat variety. Shreds from the posterior urethra are particles of secretion which have been expelled from the ducts of the prostate. They are shorter and thicker than those from the anterior urethra and usually sink rapidly to the bottom of the glass. The epithelial cells in the latter are of the transitional form, and it is difficult to differentiate them from those of the bladder, ureters and kidneys. Blood-cells in one or other glass will indicate whether the hæmorrhage is from the anterior or posterior urethra. The writer reports three cases to illustrate the practical utility of these tests. In one case a chronic discharge was shown to come from the anterior urethra and a cure was effected by using Kollmann's anterior dilator and occasional irrigations of 1 in 1,000 nitrate of silver solution. In a second case, after treating a stricture, posterior urethritis was found to exist. This was cured by posterior dilatations and regular instillations of nitrate of silver. In the third case the test showed that the source of a hæmorrhage was in the anterior urethra.

**Hydronephrosis from Papilloma of the Renal Pelvis.**—Harry B. Reynolds (San Francisco)[5] reports an interesting case of this kind. The patient, a man of 66, for nine months had observed a gradual increase in the size of the abdomen. Often he would urinate several times in the night and void a large quantity of urine. On several occasions he had passed blood-stained urine. At no time had he had pain. The abdomen became distended to the extent of that in full-term pregnancy, and a large, tense, rounded mass was felt filling the left side and extending well across the middle line. This felt like a thin walled cyst. The colon, on inflation, was found to partially lie over the swelling. There was a moderate sized double varicocele. The diagnosis seemed to lie between pancreatic cyst, supra-renal tumour, perinephric cyst, and hydronephrosis from papilloma of the renal pelvis, or a malignant tumour causing pressure on the ureter. By an operation lasting over two hours the cyst was removed. The patient died on the fifth day from hypostatic pneumonia. The cyst was a large hydronephrotic sac, with no tissue showing kidney structure. A papilloma the size of a small tomato was situated just above the ureteric orifice and obstructed its entrance. Such tumours are exceedingly rare. Albarran and Imbert, in 1903, in their exhaustive study of renal tumours, were able to collect only twenty-two cases of papilloma of the renal pelvis. The characteristic symptoms are progressive hydronephrosis, occasional bloody urine and polyuria, and absence of pain, except for occasional mild colic from the passage of clots or bits of tumour through the ureter.

**The Treatment of Accidental Wound of the Ureter.**—E. C. Dudley (Chicago)[6] reports that in performing vaginal hysterectomy for carcinoma of the body of the uterus he found the growth had extended so far forwards that the bladder had to be opened immediately in front of the cervix, and in cutting through the right broad ligament the ureter was divided. To remedy this the bladder wall was punctured from within out at the nearest point to the cut ureter through the vesico-vaginal fistula which had been made with a long slender forceps. Then, after splitting the cut end of the ureter and denuding the bladder mucosa on both sides of the puncture, the end of the ureter was drawn into the bladder and sutured there with fine cat-gut. Thus the end of the ureter was held widely open and it filled the punctured bladder wound in a water-tight manner. The vesico-vaginal fistula was closed and the vaginal wound sutured as usual in vaginal hysterectomy. The bladder was kept empty for two weeks with a self-retaining catheter. This method of affecting a uretero-cystostomy gives great security against constriction of the end of the ureter where it enters the bladder. Cystoscopy some weeks later showed a perfectly patulous ureteral opening. The author writes that he would be inclined to repeat the operation, if necessity arose, and to make an artificial vesico-vaginal-fistula for the purpose, if the bladder was not open.

[4] Med. Rec., April 2, 1904, p. 531. [5] Annals of Surgery, May 1904, p. 743. [6] Ibid., p. 755.

